# Promoter DNA recognition by the *Enterococcus faecalis* global regulator MafR

**DOI:** 10.3389/fmolb.2023.1294974

**Published:** 2023-12-13

**Authors:** Ana Moreno-Blanco, Radoslaw Pluta, Manuel Espinosa, Sofía Ruiz-Cruz, Alicia Bravo

**Affiliations:** ^1^ Centro de Investigaciones Biológicas Margarita Salas, Consejo Superior de Investigaciones Científicas (CSIC), Madrid, Spain; ^2^ Institute for Research in Biomedicine (IRB Barcelona), The Barcelona Institute of Science and Technology, Barcelona, Spain

**Keywords:** AlphaFold, *Enterococcus faecalis*, global regulators, phosphotransferase systems, protein-DNA interactions, two-component systems

## Abstract

When *Enterococcus faecalis* is exposed to changing environmental conditions, the expression of many genes is regulated at the transcriptional level. We reported previously that the enterococcal MafR protein causes genome-wide changes in the transcriptome. Here we show that MafR activates directly the transcription of the *OG1RF_10478* gene, which encodes a hypothetical protein of 111 amino acid residues. We have identified the *P10478* promoter and demonstrated that MafR enhances the efficiency of this promoter by binding to a DNA site that contains the −35 element. Moreover, our analysis of the OG1RF_10478 protein AlphaFold model indicates high similarity to 1) structures of EIIB components of the bacterial phosphoenolpyruvate:carbohydrate phosphotransferase system, and 2) structures of receiver domains that are found in response regulators of two-component signal transduction systems. However, unlike typical EIIB components, OG1RF_10478 lacks a Cys or His residue at the conserved phosphorylation site, and, unlike typical receiver domains, OG1RF_10478 lacks a conserved Asp residue at the position usually required for phosphorylation. Different from EIIB components and receiver domains, OG1RF_10478 contains an insertion between residues 10 and 30 that, according to ColabFold prediction, may serve as a dimerization interface. We propose that OG1RF_10478 could participate in regulatory functions by protein-protein interactions.

## 1 Introduction

The Gram-positive bacterium *Enterococcus faecalis* is a member of the healthy human gut microbiota. Nevertheless, if it gains access to extra-intestinal niches (*e.g.*, urinary tract, heart, or blood), it can cause deadly or serious diseases, especially in susceptible hosts (Banla et al., 2019; Kao and Kline, 2019). The opportunistic behaviour of *E. faecalis* (harmless commensal that may turn pathogenic) reflects its ability to modulate gene expression in response to specific environmental signals and host-imposed stresses (adaptive responses). This ability partially relies on proteins that act as global transcriptional regulators. In some cases, these regulators increase the activity of multiple promoters by binding to specific DNA sites that are located either upstream of or overlapping the main promoter elements (Browning and Busby, 2016; Bervoets and Charlier, 2019). Characterization of the DNA binding preferences of particular proteins has shown that some of them recognize intrinsic DNA structural characteristics (shape readout mechanism) rather than particular nucleotide sequences (base readout mechanism) (Rohs et al., 2010; Abe et al., 2015). Moreover, examples, where both direct DNA sequence readout and DNA shape recognition are required for protein binding, have also been reported (Deng et al., 2015; Ding et al., 2015; Al-Zyoud et al., 2016; Freda et al., 2023).

*E. faecalis* shows high levels of genetic diversity (Bakshi et al., 2016; He et al., 2018). The genome sequence of the well-studied OG1RF strain was published in 2008 (Bourgogne et al., 2008). Using genome-wide microarrays designed for this strain, we found that the enterococcal protein MafR (482 amino acid residues) functions as a global transcriptional regulator (Ruiz-Cruz et al., 2016). It influences positively the transcription of numerous genes, including genes that encode enzymes and transporters required for the utilization of carbon sources (*e.g.*, mannitol, glycerol, gluconate, maltose, and citrate). Later, combining *in vivo* and *in vitro* approaches, we demonstrated that MafR acts as a transcription activator (Ruiz-Cruz et al., 2019). It activates directly the transcription of two genes: *OG1RF_12294*, which is predicted to encode a calcium-transporting P-type ATPase, and *OG1RF_11486*, which encodes a putative QueT transporter family protein. In both cases, MafR stimulates transcription by binding to a DNA site that overlaps promoter sequences. Because of these results, we proposed that MafR could have a regulatory role in calcium homeostasis and queuosine synthesis (Ruiz-Cruz et al., 2019). Through bioinformatics analyses, we have shown that MafR is highly conserved among many strains whose genomes have been sequenced (Ruiz-Cruz et al., 2018). Furthermore, gel filtration chromatography and analytical ultracentrifugation studies demonstrated that MafR behaves as a dimer in solution (Ruiz-Cruz et al., 2018). *In vitro* analyses of protein-DNA interactions established that 1) MafR binds to linear double-stranded DNAs in a nonsequence-specific manner (Ruiz-Cruz et al., 2018), 2) MafR binds to DNA sites that contain regions of potential bendability (Ruiz-Cruz et al., 2019), and 3) MafR generates multimeric complexes on linear double-stranded DNAs (Ruiz-Cruz et al., 2018).

The bacterial phosphoenolpyruvate:carbohydrate phosphotransferase system (PTS) transports and phosphorylates numerous carbohydrates. It is usually composed of four cytoplasmic proteins (EI, HPr, EIIA, EIIB) and one membrane-spanning protein (EIIC). Unlike EI and HPr, the EIIA, EIIB, and EIIC components are normally specific for one substrate. To phosphorylate the carbohydrates during their transport, the cytoplasmic PTS proteins form a phosphorylation cascade starting with the phosphoenolpyruvate-mediated autophosphorylation of the EI protein (Deutscher et al., 2014). The PTS not only functions as a carbohydrate transporter but also regulates numerous cellular processes either by phosphorylating its target proteins or by interacting with them. For instance, some proteins have integrated a specific PTS-recognized phosphorylation domain known as the PTS regulation domain (PRD). PRDs have been found in both transcription antiterminators and transcription activators that control the expression of genes involved in the utilization of carbohydrates (Deutscher et al., 2014; Galinier and Deutscher, 2017). Examples where unphosphorylated or phosphorylated PTS components (HPr, EIIA, and EIIB) interact with particular proteins, including transcription regulators, have also been reported. Such interactions can either stimulate or inhibit the function of the target proteins (Galinier and Deutscher, 2017).

MafR belongs to a family of transcriptional regulators that includes Mga from *Streptococcus pyogenes* (Hondorp et al., 2013), AtxA from *Bacillus anthracis* (Hammerstrom et al., 2015), and Mga*Spn* and PclR from *S. pneumoniae* (Solano-Collado et al., 2012; Moreno-Blanco et al., 2022). This family has been defined as a new class of PRD-containing regulators that have two N-terminal helix-turn-helix DNA-binding motifs (Hondorp and McIver, 2007; Tsvetanova et al., 2007; Hondorp et al., 2013; Hammerstrom et al., 2015). In the case of Mga, both motifs were found to be required for DNA-binding and transcriptional activation (McIver and Myles, 2002; Vahling and McIver, 2006). Moreover, the C-terminal region of the members of this family is predicted to have structural homology to PTS-EIIB components (EIIB-like domain). In AtxA and Mga, the C-terminal region was shown to be involved in self-association (Hammerstrom et al., 2011; Hondorp et al., 2012).

The two-component signal transduction systems (TCSs) play important roles in bacterial colonization and adaptation to new niches. In a prototypical TCS, the response regulator generates a specific cellular response to the signal detected by the cognate histidine kinase (Gao et al., 2019). Usually, the response regulators have a receiver (REC) domain and an effector domain. The histidine kinase phosphorylates the REC domain on a conserved Asp residue. In a majority of response regulators, the effector domain is a DNA-binding domain, however, response regulators having an RNA-binding domain or a protein-binding domain have also been reported. Still more, there are examples of response regulators that lack an effector domain (known as single-domain response regulators) (Galperin, 2006; Jenal and Galperin, 2009). Some of the latter regulators are unclassified because their encoding genes are not located in the vicinity of histidine kinase-encoding genes (Nguyen et al., 2015).

The enterococcal *OG1RF_10478* gene (new locus_tag OG1RF_RS02540) encodes a hypothetical protein of 111 amino acid residues (NCBI Reference Sequence WP_002358719.1). In this study, we demonstrate that MafR activates directly the transcription of this gene. We show that, in exponentially growing bacteria, the RNA polymerase recognizes the *P10478* promoter to initiate transcription of the *OG1RF_10478* gene. The activity of this promoter is enhanced in the presence of the MafR regulator. We also show that MafR interacts with a region that contains both the −35 element of the *P10478* promoter and a site that is needed for its activation by MafR. Furthermore, we have obtained an AlphaFold three-dimensional model of the OG1RF_10478 protein and found that it has similarity to 1) structures of PTS-EIIB components, and 2) structures of REC domains present in response regulators of TCSs. We discuss that protein OG1RF_10478 could have a regulatory role.

## 2 Materials and Methods

### 2.1 Oligonucleotides, bacterial strains, and plasmids

The oligonucleotides used in this work are listed in Table 1. *E. faecalis* strains OG1RF (Bourgogne et al., 2008) and OG1RF∆*mafR* (Ruiz-Cruz et al., 2016) were used. OG1RF∆*mafR* lacks the *mafR* regulatory gene. Plasmid pDLF is a constitutive expression vector based on the enterococcal promoter *P2493* (Ruiz-Cruz et al., 2010; Ruiz-Cruz et al., 2016). It carries a kanamycin resistance gene. Plasmid pDLF*mafR* is a pDLF derivative that carries the *P2493*::*mafR* fusion gene (Ruiz-Cruz et al., 2016). Plasmid pASTT is a promoter-probe vector based on the *gfp* reporter gene (Ruiz-Cruz et al., 2019). It carries a tetracycline resistance gene. The following pASTT-derivatives were constructed in this work. In all cases, a region of the OG1RF chromosome was amplified by PCR using the indicated primers. Then, the PCR product was digested with *Sac*I, and the restriction fragment was ligated to the *Sac*I-linearized pASTT vector: (a) pASTT-*P10478* (primers F*10478* and R*10478*, 301-bp restriction fragment), (b) pASTT-*P10478∆122* (primers F*10478∆122* and R*10478*, 177-bp restriction fragment), (c) pASTT-*P10478∆210* (primers F*10478∆210* and R*10478*, 90-bp restriction fragment), (d) pASTT-*P10478∆228* (primers F*10478∆228* and R*10478*, 73-bp restriction fragment), (e) pASTT-*P10478∆234* (primers F*10478∆234* and R*10478*, 67-bp restriction fragment), (f) pASTT-*P10478∆243* (primers F*10478∆243* and R*10478*, 58-bp restriction fragment), and (g) pASTT-*P10478∆-10* (primers F*10478* and R*10478∆-10*, 279-bp restriction fragment). For overproduction of MafR-His, an inducible expression system based on the *Escherichia coli* strain BL21 (DE3) (a gift of F. W. Studier) and the pET24b-*mafR*-His plasmid (Ruiz-Cruz et al., 2018) was used. This plasmid encodes the MafR-His protein, which carries the Leu-Glu-6xHis peptide fused to its C-terminus.

**TABLE 1:** Oligonucleotides used in this work.

Name	Sequence (5′to 3′)^a^
F*10478*	CAC​TAA​TAC​AG**A**G**C**T**C** AAT​GTT​GTC​A
R*10478*	GCA​TAC​GCT​TA **G**A**G**CTCCTA​GAA​CT
F*10478∆122*	CTG​TTT​CCA​GTG​A **G**AG**C**TCTAT​TGT​ACC​T
F*10478∆210*	GAT​AGT​TTA​AAA​C **G**A**G**C**T**CCAA​GCG​TTG​T
F*10478∆228*	GAT​AGT​TTA​AAA​CTA​TCA​CCA​AGC​GTT​GTT **GAG**CT**C** GTC​ACT​GT
F*10478∆234*	GAT​AGT​TTA​AAA​CTA​TCA​CCA​AGC​GTT​GTT​TTA​CTAG**AGC​TC** GTA​AAA​A
F*10478∆243*	CTA​GTC​ACT​GTA **G**A**GC**T**C** ATT​TGT​TTT​T
R*10478∆-10*	GAA​CTA​AGT​ATA​C **G**A**GC**TCAGC​TTA
F*recA*-q	GCA​ACG​AAA​TGG​TGG​AAC​AG
R*recA*-q	AAG​GCA​TCG​GCA​ATC​TCT​AAG
F*10478*-q	GAG​CAG​CAA​AAC​ATC​AAA​GCC​T
R*10478*-q	AGA​TAG​GGA​GCG​AGC​ATT​TC
F*10478*-D	GCA​AAC​TGT​TTC​CAG​TGA​TAG​T
R*10478*-D	CCA​CAA​ACA​ACG​TCG​CTC​CAT​CA
F*10478*-S	GTC​ATT​TTA​GTT​CCT​CCC​TAT​GT
R*10478*-S	GGC​TTT​GAT​GTT​TTG​CTG​CTC​T

^a^ Restriction sites are underlined, and the base changes that generate restriction sites are in bold.

### 2.2 Growth and transformation of bacteria

The *E. faecalis* strains were grown in Bacto™ Brain Heart Infusion (BHI) medium, at 37°C in a static water bath. For plasmid-harbouring strains, the medium was supplemented with kanamycin (250 μg/mL; pDLF and its derivatives) and/or tetracycline (4 μg/mL; pASTT and its derivatives). The *E. coli* strain BL21 (DE3) harbouring plasmid pET24b-*mafR*-His was grown in tryptone-yeast extract (TY) medium supplemented with kanamycin (30 μg/mL), at 37°C in a shaking water bath. The protocol used to transform *E. faecalis* by electroporation was described (Shepard and Gilmore, 1995).

### 2.3 DNA and RNA isolation

Genomic DNA was prepared using the Bacterial Genomic Isolation Kit (Norgen Biotek Corporation). Plasmid DNA was prepared using the High Pure Plasmid Isolation Kit (Roche Applied Science) as described (Ruiz-Cruz et al., 2016). Total RNA was isolated using the RNeasy Mini Kit (QIAGEN) as reported (Ruiz-Cruz et al., 2016). The integrity of rRNAs was analysed by agarose gel electrophoresis. RNA concentration was determined using a NanoDrop ND-2000 Spectrophotometer.

### 2.4 Polymerase chain reaction

The Phusion High-Fidelity DNA polymerase (Thermo Scientific) and the Phusion HF buffer were used as described (Ruiz-Cruz et al., 2016). PCR products were purified using the QIAquick PCR Purification Kit (QIAGEN).

### 2.5 Quantitative reverse transcription PCR

For each strain, total RNA was isolated from three independent bacterial cultures. Then, from each RNA preparation, cDNA was synthesized using random primers and the iScript Select cDNA Synthesis Kit (Bio-Rad) as described (Ruiz-Cruz et al., 2016). To rule out the presence of genomic DNA in the RNA preparations, reactions without adding reverse transcriptase were performed. Quantitative PCRs were carried out using the iQ SYBR Green Supermix (Bio-Rad) and an iCycler Thermal Cycler (Bio-Rad) as reported (Ruiz-Cruz et al., 2016). From each cDNA sample, three PCRs per gene (gene of interest and internal control gene) were performed. Data were analysed with the iQ™5 Optical System Software. Relative quantification of gene expression was performed using the comparative *C*_T_ method (Schmittgen and Livak, 2008) as described (Moreno-Blanco et al., 2022). The *recA* gene (*OG1RF_12439*; recombination protein RecA) was used as the internal control gene (oligonucleotides F*recA*-q and R*recA*-q). The oligonucleotides F*10478*-q and R*10478*-q (Table 1) were used to determine the relative expression of the *OG1RF_10478* gene. The threshold cycle values (*C*_T_) of *OG1RF_10478* and *recA* were used to calculate 2^−Δ*C*_T_^, where Δ*C*_T_ = *C*_T_ *OG1RF_10478*-*C*_T_ *recA*. For the *OG1RF_10478* gene, the fold change in expression (FC) in one strain compared to another was obtained by dividing the corresponding mean 2^−Δ*C*_T_^ values as reported (Moreno-Blanco et al., 2022).

### 2.6 Primer extension

The oligonucleotide R*10478*-D was radioactively labelled at the 5′-end using [γ-^32^P]-ATP (PerkinElmer) and T4 polynucleotide kinase (New England Biolabs) as reported (Solano-Collado et al., 2013). Primer extension reactions were performed using total RNA from the indicated bacterial strain, the ^32^P-labelled oligonucleotide R*10478*-D, and the ThermoScript Reverse Transcriptase enzyme (Invitrogen) as described (Ruiz-Cruz et al., 2019). cDNA products were analysed by sequencing gel (8 M urea, 6% polyacrylamide) electrophoresis. Dideoxy-sequencing reactions (Sequenase Version 2.0 DNA Sequencing Kit; USB Corporation) were run in the same gel as DNA size markers. These reactions were carried out using a 422-bp PCR-amplified DNA fragment (OG1RF DNA and oligonucleotides F*10478*-S and R*10478*-S) as DNA template and the ^32^P-labelled oligonucleotide R*10478*-D as primer. Labelled products were visualized using a Fujifilm Image Analyser FLA-3000.

### 2.7 Fluorescence assays

Plasmid-harbouring enterococcal strains were grown as indicated above to an optical density at 650 nm (OD_650_) of 0.4 (exponential phase). Then, different volumes of culture (0.4–1 mL) were centrifuged. Cells were resuspended in 200 µL of phosphate-buffered saline (PBS). For each strain, three independent cultures were analysed. Fluorescence intensity was measured using a Thermo Scientific Varioskan Flash instrument (excitation at 488 nm and emission at 515 nm).

### 2.8 Purification of MafR-His

The protocols used to overproduce and purify the MafR-His protein were described previously (Ruiz-Cruz et al., 2018). Purification of this protein involved the use of fast-pressure liquid chromatography (Biologic Duoflow, Bio-Rad) on a nickel affinity column (HisTrap HP column, GE Healthcare). Protein concentration was determined using a NanoDrop ND-2000 Spectrophotometer (Thermo Scientific).

### 2.9 DNase I footprinting assays

A 266-bp region of the OG1RF genome (coordinates 498475 to 498210) was amplified by PCR using the oligonucleotides F*10478*-D and R*10478*-D (Table 1). To ^32^P-label the amplified DNA fragment at either the 5′-end of the coding strand or the 5′-end of the non-coding strand, either the oligonucleotide F*10478*-D or the oligonucleotide R*10478*-D was ^32^P-labelled at the 5′-end as described (Solano-Collado et al., 2013). Binding reactions and DNase I digestion were performed as reported (Ruiz-Cruz et al., 2019). Briefly, as shown by our previous results (Ruiz-Cruz et al., 2018), MafR (and MafR-His) binds to its primary DNA site and then additional MafR units bind sequentially to the same DNA molecule, generating multiple complexes. Therefore, in the binding reactions, and previous to DNA digestion with DNase I, free DNA molecules and various types of MafR-DNA complexes coexist. Their proportion will depend on the concentration of MafR. To favour the presence of complexes constituted by MafR bound to its primary site versus higher-order MafR-DNA complexes, we have to work with low protein concentrations and, under such conditions, the amount of free DNA molecules in the reaction mixtures can be high. Samples were analysed by sequencing gel (8 M urea, 6% polyacrylamide) electrophoresis. Dideoxy-sequencing reactions (Sequenase Version 2.0 DNA Sequencing Kit; USB Corporation) were run in the same gel. These reactions were carried out using a 422-bp PCR-amplified DNA fragment (OG1RF DNA and oligonucleotides F*10478*-S and R*10478*-S) as DNA template and either the ^32^P-labelled oligonucleotide F*10478*-D (sequence of the coding-strand relative to the *P10478* promoter) or the ^32^P-labelled oligonucleotide R*10478*-D (sequence of the non-coding-strand) as primer. Labelled products were visualized using a Fujifilm Image Analyser FLA-3000 and the intensity of the bands was quantified using the Quantity One software (Bio-Rad). Briefly, to compare the lane corresponding to “300 nM MafR-His” with the lane corresponding to “no MafR-His,” we determined the relative intensity of each band detected at “300 nM MafR-His” with respect to the same band in the “no MafR-His” lane. Then, we compared the relative intensities of all the bands corresponding to “300 nM MafR-His” and we detected that some of the bands had a lower relative intensity. Such bands define regions with changes in the relative sensitivity to DNase I (diminished cleavages; regions protected against DNase I digestion). This is a quantitative measurement that eliminates the differences in loading and defines the primary binding site of MafR.

### 2.10 Electrophoretic mobility shift assays

Binding reactions were performed as described (Ruiz-Cruz et al., 2018). The ^32^P-labelled 266-bp DNA fragment (4 nM) was incubated with different amounts of MafR-His. Reaction mixtures were analysed by electrophoresis on native polyacrylamide (6%) gels. Labelled DNA was visualized using a Fujifilm Image Analyser FLA-3000.

### 2.11 Bioinformatics analyses

The bendability/curvature propensity plot was calculated with the bend it server (http://pongor.itk.ppke.hu/dna/bend_it.html) (Vlahovicek et al., 2003). The BPROM bacterial σ70-dependent promoter prediction program (*Softberry, Inc.*) (http://www.softberry.com) was used. AlphaFold Protein Structure Database (Jumper et al., 2021; Varadi et al., 2022) and RoseTTAFold (Baek et al., 2021) were used to obtain three-dimensional models of the OG1RF_10478 protein (Uniprot Q837T7). The Dali server was employed for a search of similar structures (Holm, 2022). The ColabFold accelerated prediction of protein structures and complexes software (https://colab.research.google.com/github/sokrypton/ColabFold/blob/main/AlphaFold2.ipynb) (Mirdita et al., 2022) was used to explore the dimerization potential of OG1RF_10478. Figures, representing structural findings, were either directly taken from the abovementioned resources or generated using UCSF Chimera (Pettersen et al., 2004) and PyMol (*Schrödinger, Inc.*).

## 3 Results

### 3.1 MafR has a positive effect on the transcription of the *OG1RF_10478* gene

**FIGURE 1:**
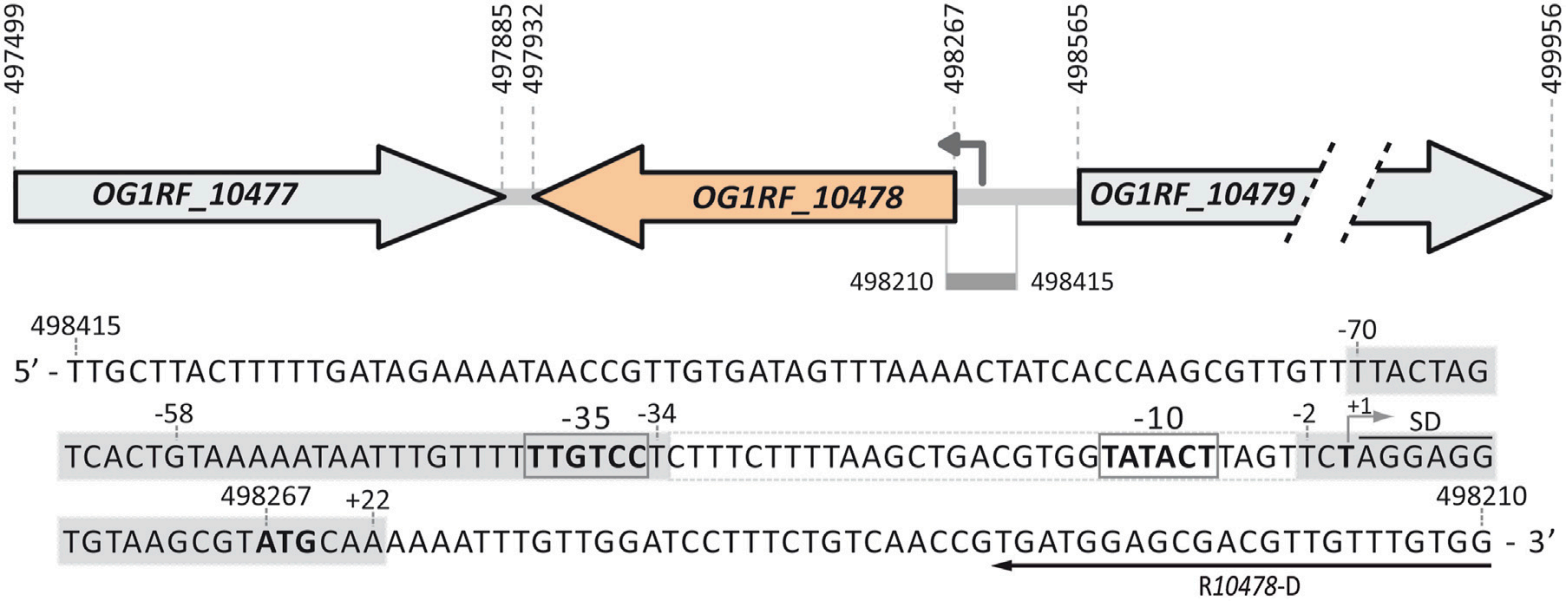
Relevant features of the *P10478* promoter region. Gene *OG1RF_10477* (new locus_tag OG1RF_RS02535) encodes a transposase (128 residues, NCBI Reference Sequence WP_002358717.1). Gene *OG1RF_10478* (new locus_tag OG1RF_RS02540) encodes a hypothetical protein (111 residues, WP_002358719.1). Gene *OG1RF_10479* (new locus_tag OG1RF_RS02545) encodes a sodium/dicarboxylate symporter family protein (463 residues, WP_002355598.1). For each gene, the coordinates of the translation start and stop codons are indicated. The arrow upstream of the *OG1RF_10478* gene represents the *P10478* promoter identified in this work. The nucleotide sequence of the region spanning coordinates 498415 and 498210 of the *E. faecalis* OG1RF genome (GenBank CP002621.1) is shown. The −35 and −10 elements of the *P10478* promoter are indicated. The transcription start site (+1 position; this work), the putative Shine-Dalgarno sequence (SD), and the translation start codon (ATG in boldface) of the *OG1RF_10478* gene are indicated. The position of the R*10478*-D oligonucleotide used for primer extension is shown. The grey boxes denote the MafR-His binding sites defined by DNase I footprinting assays (this work).

**FIGURE 2:**
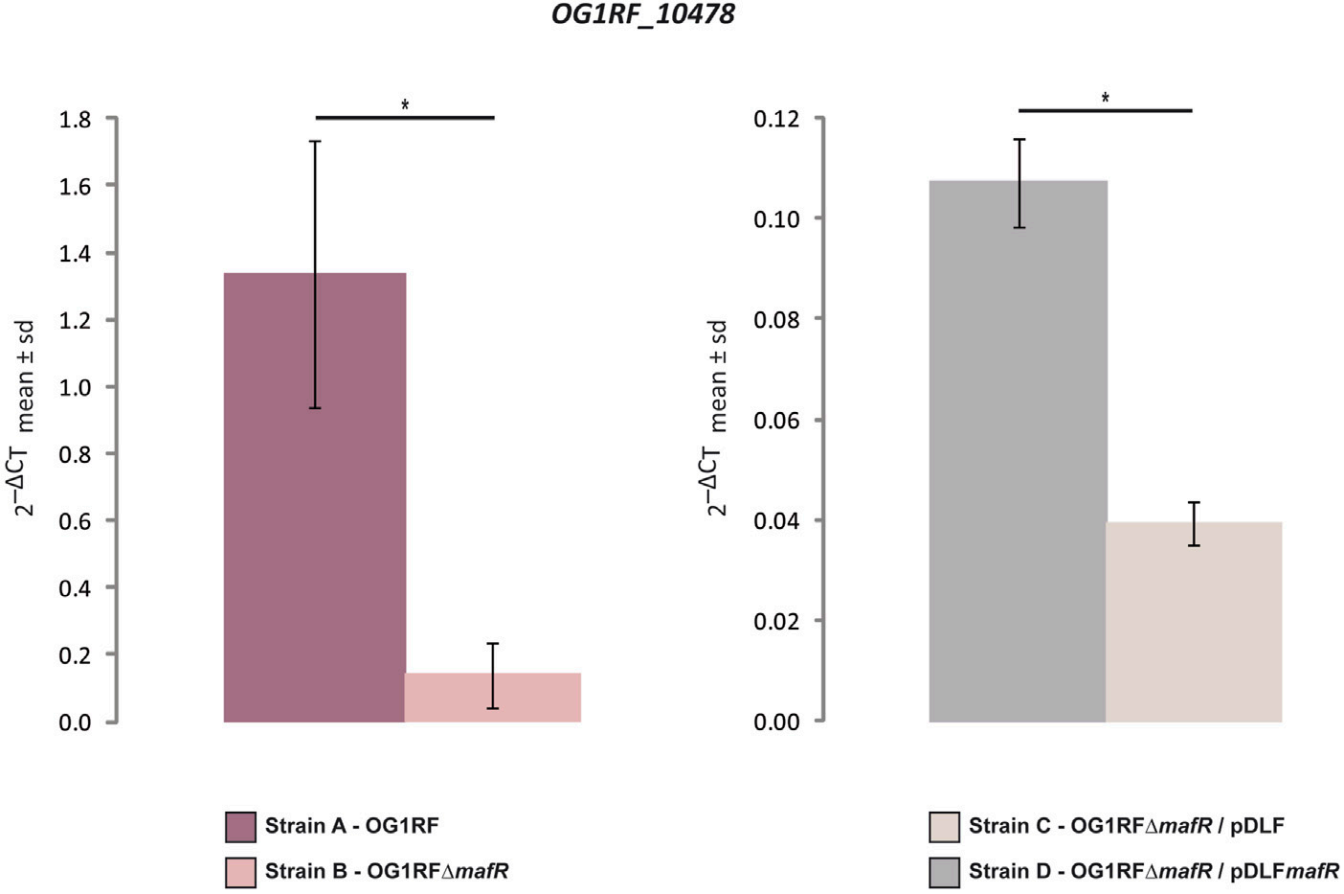
Effect of MafR on the transcription of the *OG1RF_10478* gene. The relative expression of the *OG1RF_10478* gene was determined in the indicated strains by qRT-PCR. The graphical representation shows the mean ± standard deviation (sd) of the 2^−ΔC_T_^ values, which were calculated from the data shown in Supplementary Tables S1, S2. *p*-values were calculated using the Student´s *t*-test (paired, two-tailed); *p < 0.05.

Our previous work showed that the MafR protein of *E. faecalis* causes genome-wide changes in the transcriptome (Ruiz-Cruz et al., 2016). Specifically, by DNA microarray studies, we compared the transcriptional profiles of the *E. faecalis* strains OG1RF (wild-type) and OG1RF∆*mafR* (*mafR* deletion mutant) grown to mid-log phase under standard laboratory conditions (BHI broth, 37°C, without aeration). This analysis revealed that MafR activates the transcription of numerous genes, either directly or indirectly (Ruiz-Cruz et al., 2016). Among them, transcription of the *OG1RF_10478* gene (Figure 1), which encodes a hypothetical protein of 111 amino acid residues, was found to be higher (∼7-fold) in the wild-type strain. In this work, we addressed the validation of such a finding by quantitative RT-PCR (qRT-PCR) assays using the comparative *C*_T_ method (Schmittgen and Livak, 2008). First, we determined the relative expression of the *OG1RF_10478* gene in the strains OG1RF and OG1RF∆*mafR* using the *recA* gene as a reference gene (Figure 2 and Supplementary Table S1). The amount of *OG1RF_10478* transcripts was higher (∼9.6-fold) in the wild-type strain. Next, we determined the relative expression of the *OG1RF_10478* gene in two plasmid-harbouring strains that lack the chromosomal *mafR* gene: strain OG1RF∆*mafR*/pDLF (absence of MafR) and strain OG1RF∆*mafR*/pDLF*mafR* (plasmid-encoded MafR) (Figure 2 and Supplementary Table S2). The amount of *OG1RF_10478* transcripts was higher (∼2.7-fold) in the strain OG1RF∆*mafR*/pDLF*mafR*, indicating that plasmid-encoded MafR has a positive effect on the transcription of the *OG1RF_10478* gene under laboratory conditions.

### 3.2 MafR enhances the activity of the *P10478* promoter

**FIGURE 3:**
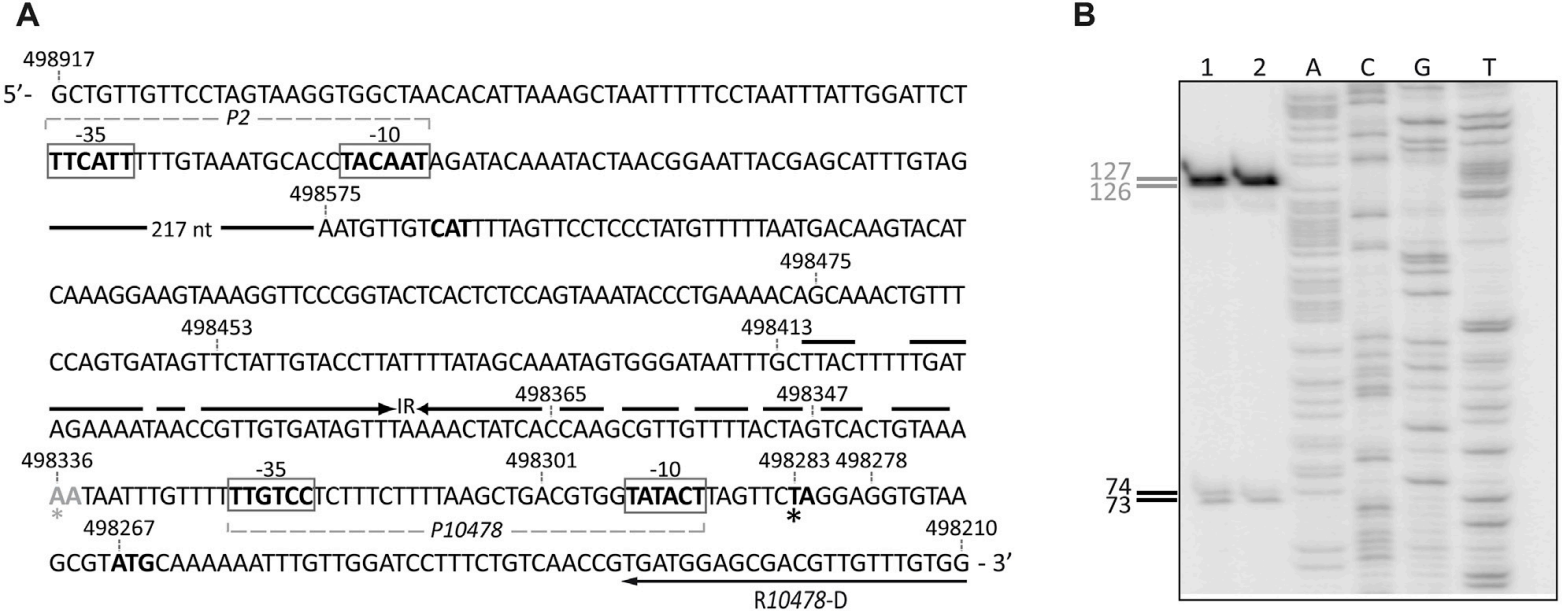
Primer extension reactions. **(A)** Nucleotide sequence of the region spanning coordinates 498917 and 498210 of the OG1RF genome. The −35 and −10 elements of the *P2* and *P10478* promoters are indicated. The translation start codon of the *OG1RF_10478* gene (ATG in boldface, coordinate 498267) is shown. The imperfect inverted repeat (IR) is indicated. The position of the R*10478*-D oligonucleotide used for primer extension is shown. The black and grey asterisks denote the position of the 3′-end in cDNA products of 74- and 127-nt, respectively. **(B)** Primer extension reactions were carried out using total RNA from strain OG1RF∆*mafR*/pDLF*mafR* (lane 1) and strain OG1RF (lane 2). The R*10478*-D oligonucleotide was used as a primer. Dideoxy-mediated chain termination sequencing reactions (see Materials and Methods) were run in the same gel as DNA size markers (lanes A, C, G, and T). The size (in nt) of the cDNA products is indicated on the left of the gel.

The bacterial RNA polymerase core enzyme is a multi-subunit complex. It minimally consists of five subunits (α_2_ββ´ω). However, a large number of bacteria, and particularly the Firmicutes, contain additional small subunits (δ and ε) (Weiss and Shaw, 2015). The RNA polymerase core enzyme interacts transitory with one of a set of sigma factors forming the RNA polymerase holoenzyme. The promoter specificity of the RNA polymerase depends on its sigma factor. In exponentially growing bacterial cells, the majority of promoters are recognized by the housekeeping sigma factor (known as σ70 in *E. coli* and SigA in *E. faecalis*) (for a review see Browning and Busby, 2016). According to the BPROM bacterial σ70-dependent promoter prediction program (*Softberry, Inc.*), the region of 650 nucleotides (nt) immediately upstream of the translation start site (coordinate 498267) of the *OG1RF_10478* gene has two potential σ70-dependent promoters, here named *P10478* (proximal promoter) and *P2* (distal promoter) (Figure 3A). Compared to the consensus sequence of the promoters recognized by the *E. coli* σ70 factor, the proximal promoter *P10478* has a near-consensus −10 element (5′-TATAcT-3′) and shows a 4/6 match at the −35 element (5′-TTGtCc-3′). The spacer length between the two promoter elements is 22-nt, the optimal length being 17-nt. Moreover, the *P10478* promoter has a 5′-TG-3′ motif positioned one base upstream of the −10 element (the extended −10 element). In Gram-positive bacteria, numerous promoters have the 5′-TRTG-3′ motif (R = purine) located at such a position (Voskuil and Chambliss, 1998; Voskuil and Chambliss, 2002; Solano-Collado et al., 2021). The distal promoter *P2* has also a near-consensus −10 element (5′-TAcAAT-3′) and shows a 3/6 match at the −35 element (5′-TTcAtt-3′). This predicted promoter has also non-optimal spacing (14-nt) between the two elements.

To identify transcription initiation sites of the *OG1RF_10478* gene, we performed primer extension assays using the oligonucleotide R*10478*-D as a primer (Figure 3A). To favour the presence of *OG1RF_10478* transcripts in the RNA preparations, we isolated total RNA from enterococcal cells that synthesize the MafR regulator: strain OG1RF (chromosome-encoded MafR) (Figure 3B, lane 2) and strain OG1RF∆*mafR*/pDLF*mafR* (plasmid-encoded MafR) (Figure 3B, lane 1). In both primer extension reactions, 73-, 74-, 126-, and 127-nt cDNA products were detected. The 73- and 74-nt cDNA products could be generated by reverse transcriptase running off at 5′ ends of transcripts initiated at coordinates 498282 and 498283, respectively (Figure 3A). These coordinates are located 8- and 7-nt downstream of the −10 element of the *P10478* promoter. The 126- and 127-nt cDNA products could correspond to transcription initiation events at coordinates 498335 and 498336, respectively (Figure 3A). However, a σ70-dependent promoter just upstream of such coordinates was not predicted, and could not be detected by visual inspection of the sequence. Alternatively, since there is an imperfect inverted repeat between coordinates 498336 and 498413 (Figure 3A), reverse transcriptase pausing at potential RNA structures could generate the 126- and 127-nt cDNAs. Thus, these results suggested that the enterococcal RNA polymerase could recognize two promoters to initiate transcription of the *OG1RF_10478* gene: the *P10478* promoter and a promoter located upstream of the coordinate 498336. The latter promoter could be either *P2* or another undetected promoter. This work has been focused on the characterization of the *P10478* promoter (see below). The transcription start site mapped at coordinate 498282 of the OG1RF genome (promoter *P10478*) coincides with the transcription start site mapped at coordinate 699386 of the *E. faecalis* V583 genome (Michaux et al., 2020). The latter transcription start site was mapped using the dRNA-seq technique in combination with ANNOgesic analysis and was associated with the *EF0743* gene (*OG1RF_10478* in strain OG1RF).

**FIGURE 4:**
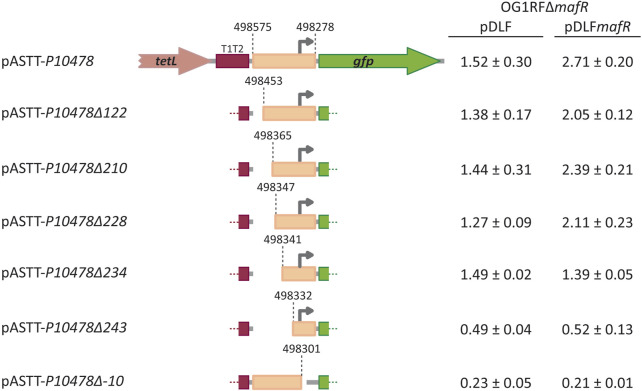
Fluorescence assays. Seven regions of the OG1RF genome were amplified by PCR and inserted into the *Sac*I site of the promoter-probe vector pASTT, which derives from pAST (Ruiz-Cruz et al., 2010; Ruiz-Cruz et al., 2019). The coordinates of the inserted regions are indicated. The *tetL* gene confers resistance to tetracycline. The T1T2 box represents the tandem transcriptional terminators T1 and T2 of the *E. coli rrnB* ribosomal RNA operon. The promoter-less *gfp* gene encodes a variant of the green fluorescent protein. The grey arrow represents the −10 element of the *P10478* promoter. Each pASTT derivative was introduced into strain OG1RF∆*mafR*/pDLF (absence of MafR) and strain OG1RF∆*mafR*/pDLF*mafR* (plasmid-encoded MafR). The intensity of fluorescence (arbitrary units) corresponds to 0.8 mL culture (OD_650_ = 0.4). In each case, three independent cultures were analysed.

Further studies using promoter-reporter fusions (Figure 4) allowed us to conclude that protein MafR activates the transcription of the *OG1RF_10478* gene from the *P10478* promoter. Specifically, the 298-bp region spanning coordinates 498278 and 498575 (see Figure 3A) was inserted into the multicopy promoter-probe vector pASTT, which is based on the *gfp* reporter gene. The recombinant plasmid (pASTT-*P10478*) was then introduced into OG1RF (chromosome-encoded MafR) and OG1RF∆*mafR* (absence of MafR). In both strains, the expression of *gfp* (1.54 ± 0.23 and 1.60 ± 0.16 units of fluorescence, respectively) was higher (∼4-fold) than the basal level (OG1RF harbouring pASTT, 0.38 ± 0.02 units). These results indicated that 1) the 298-bp region contains a promoter sequence, and 2) the chromosomal copy of *mafR* does not enhance the efficiency of such a promoter when it is located on a multicopy plasmid. However, different results were obtained when plasmid pASTT-*P10478* was introduced into OG1RF∆*mafR*/pDLF (absence of MafR) and OG1RF∆*mafR*/pDLF*mafR* (plasmid-encoded MafR) (Figure 4). The expression of *gfp* was higher (∼1.8-fold) in OG1RF∆*mafR*/pDLF*mafR*, indicating that the amount of MafR provided by pDLF*mafR* increases the activity of the promoter that drives the expression of *gfp* in plasmid pASTT-*P10478*. An additional deletion analysis (Figure 4, see also Figure 3A) showed that 1) deletion of the 24-bp region spanning coordinates 498278 and 498301 (−10 element of the *P10478* promoter) eliminates the promoter activity of the 298-bp region (plasmid pASTT-*P10478∆-10*), 2) the 70-bp region spanning coordinates 498278 and 498347 contains both the promoter and the site required for its activation by MafR (plasmids pASTT-*P10478∆122*, pASTT-*P10478∆210*, and pASTT-*P10478∆228*), 3) the 7-bp region spanning coordinates 498341 and 498347 is essential for MafR-mediated activation of the promoter (plasmid pASTT-*P10478∆234*), and 4) deletion of the 10-bp region spanning coordinates 498332 and 498341 reduces to some extent the promoter activity (plasmid pASTT-*P10478∆243*), which suggests that the A+T-rich sequence located upstream of the −35 element of the *P10478* promoter could act as an UP element (see Figure 3A). The UP element is known to stimulate promoter activity by interacting with the RNA polymerase α-subunit (Estrem et al., 1998). Taken together, we conclude that MafR has a positive effect on the activity of the *P10478* promoter in bacteria growing under laboratory conditions.

### 3.3 MafR binds to the *P10478* promoter region

By EMSA, we have previously analysed the interaction of the MafR regulator with various linear double-stranded DNAs, including DNAs without apparent sequence similarities (Ruiz-Cruz et al., 2018). On all tested DNAs, and like the Mga*Spn* regulator (Solano-Collado et al., 2013), MafR generated multimeric complexes. This study indicated that 1) multiple MafR units (likely dimers) bind sequentially to the DNA molecule, and 2) MafR binds to linear double-stranded DNAs in a manner that is not sequence-specific.

**FIGURE 5:**
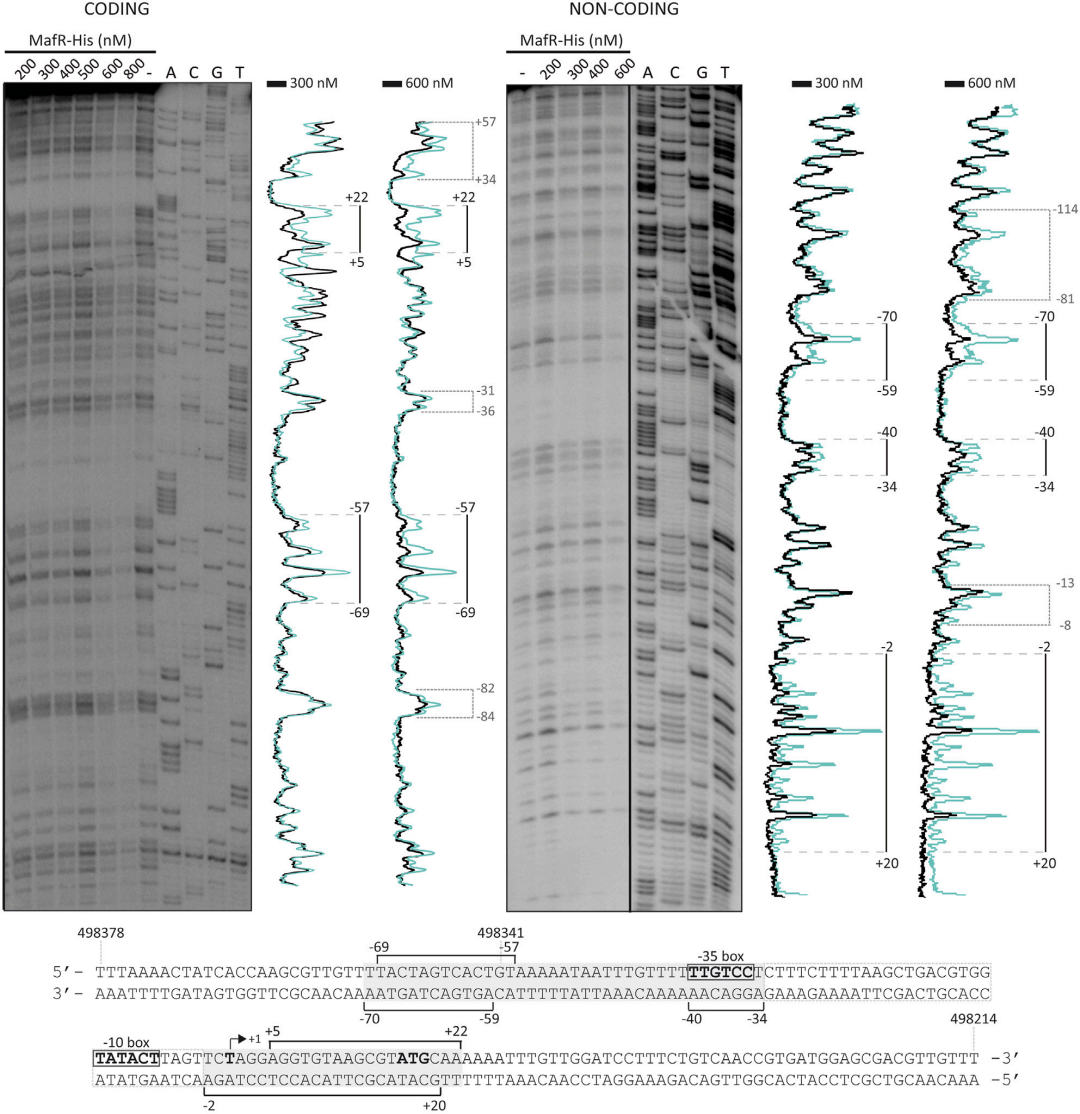
DNase I footprints of MafR-His-DNA complexes. The 266-bp DNA fragment (coordinates 498475 to 498210) was ^32^P-labelled either at the 5′-end of the coding strand or at the 5′-end of the non-coding strand. The labelled DNA (4 nM) was incubated with the indicated concentrations of MafR-His and then digested with DNase I. Dideoxy-mediated chain termination sequencing reactions were run in the same gel (lanes A, C, G, T). On the left gel, the sequence corresponds to the coding strand of the 266-bp DNA fragment (^32^P-labelled F*10478*-D oligonucleotide). On the right gel, the sequence corresponds to the non-coding strand of the 266-bp DNA fragment (^32^P-labelled R*10478*-D oligonucleotide). All the lanes displayed came from the same gel (delineation with dividing lines). Densitometer scans corresponding to DNA without MafR-His (blue line) and DNA with MafR-His (300 nM and 600 nM, black line) are shown. The protected regions are indicated with brackets. The indicated positions are relative to the transcription initiation site of the *OG1RF_10478* gene. The nucleotide sequence of the region spanning coordinates 498378 and 498214 is shown. The −35 and −10 elements of the *P10478* promoter are indicated. The transcription initiation site (+1 position) and the translation start codon (ATG) of the *OG1RF_10478* gene are shown. Brackets indicate regions protected against DNase I digestion. The grey boxes denote the MafR-His binding sites.

To analyse whether MafR recognized preferentially a site (primary binding site) on the *P10478* promoter region, we performed DNase I footprinting experiments. We used a His-tagged version of the MafR protein (MafR-His) and a 266-bp DNA fragment (coordinates 498475 to 498210; see Figure 3A), which contains both the *P10478* promoter and the sequence that is essential for its activation by MafR (coordinates 498341 to 498347; positions −58 to −64 of the *P10478* promoter) (see Figure 1, 4). The presence of a His-tag at the C-terminal end of MafR does not affect its DNA-binding properties (Ruiz-Cruz et al., 2018). The 266-bp DNA fragment was radioactively labelled either at the 5′-end of the coding strand or at the 5′-end of the non-coding strand, and the labelled DNA was then incubated with increasing concentrations of MafR-His (Figure 5). Storage phosphor imaging technology was used for quantitative comparisons of the radioactively labelled products (see Materials and Methods). On the coding strand and at 300 nM of MafR-His, changes in DNase I sensitivity (diminished cleavages) were observed from position −69 to −57, and from position +5 to +22 relative to the transcription initiation site of the *P10478* promoter. On the non-coding strand and at 300 nM of MafR-His, diminished cleavages were observed from position −70 to −59, from −40 to −34, and from −2 to +20. Therefore, protected sequences against DNase I digestion were observed mainly in two regions: between positions −70 and −34 (region A) and between positions −2 and +22 (region B). Region A includes both the −35 element of the *P10478* promoter and the sequence that is essential for its activation by MafR (positions −58 to −64). Region B is located just downstream of the −10 element. On both DNA strands and at 600 nM of MafR-His, regions protected against DNase I digestion were observed along the DNA fragment, which suggested that, upon binding to the primary site, additional MafR-His units interacted with the adjacent DNA regions. This result is consistent with the ability of MafR-His to generate multimeric complexes on linear double-stranded DNAs (Ruiz-Cruz et al., 2018). Specifically, we performed EMSA experiments with the same 266-bp DNA fragment that was used in the DNase I footprinting assays (Supplementary Figure S1). The radioactively labelled DNA was incubated with different concentrations of MafR-His. At 300 nM of MafR-His, free DNA and four protein-DNA complexes were detected. Moreover, as the protein concentration was increased, such complexes disappeared and higher-order complexes appeared.

**FIGURE 6:**
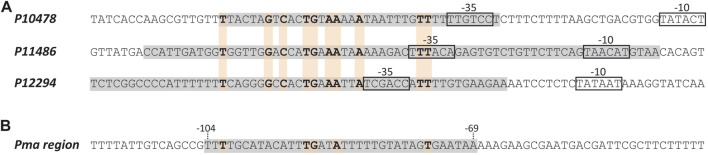
MafR binding sites. **(A)** Nucleotide sequence alignment of DNA sites recognized by MafR on three promoter regions. Such MafR binding sites are indicated with grey boxes. They have been defined by DNAse I footprinting assays (Ruiz-Cruz et al., 2019; and this work). The −35 and −10 elements of each promoter are shown. Identical nucleotides are highlighted in boldface. **(B)** Nucleotide sequence of the DNA site recognized by MafR on the *Pma* promoter region (grey box, positions −69 to −104) (Ruiz-Cruz et al., 2018). Nucleotides shared with the MafR binding sites shown in **(A)** are indicated in boldface.

Using the bend.it server (pongor.itk.ppke.hu/dna/bend_it.html) (Vlahovicek et al., 2003), we calculated the bendability/curvature propensity plot of the 266-bp DNA fragment. As shown in Supplementary Figure S2, the profile contains regions of potential bendability around the −10 element of the *P10478* promoter. Figure 6 shows a nucleotide sequence alignment of the regions recognized by MafR on the *P10478* (region A, this work), *P11486*, and *P12294* (Ruiz-Cruz et al., 2019) promoters. The three regions, defined by DNase I footprinting assays, contain the −35 promoter element. This alignment revealed that the three MafR binding sites have a low sequence identity: they share the motif **TG** (T/A)**AA** (A/T)(A/T)**A**.

### 3.4 Bioinformatics analysis of protein OG1RF_10478

In the OG1RF genome (Bourgogne et al., 2008), the ATG codon at coordinate 498267 is likely the translation start site of the *OG1RF_10478* gene (Figure 1). Translation from this codon, which is preceded by a canonical ribosome-binding site (AGGAGG), would generate a protein of 111 amino acids (NCBI Reference Sequence WP_002358719.1). Using the BLASTP protein sequence alignment program (Altschul et al., 1997), we were unable to predict the biological function of the OG1RF_10478 protein. However, the use of additional bioinformatics tools (see below) has allowed us to establish that the predicted three-dimensional structure of the OG1RF_10478 protein is highly similar to 1) structures of PTS-EIIB components, and 2) structures of REC domains present in response regulators of TCSs.

The EIIB component of a PTS is usually specific for one substrate or, in a few cases, for a small group of closely related carbohydrates (Deutscher et al., 2014). The PTS-EIIB components are phosphorylated at a conserved residue located at the end of or right after the β1-strand (residue around position number 10). In most cases, the role of phosphoryl group acceptor is played by a Cys residue, except for the EIIB components of the mannose PTS family, in which this role is played by a His residue. The phosphorylated EIIB component transfers its phosphoryl group to a carbohydrate molecule bound to the cognate EIIC component (Deutscher et al., 2014). According to the HHpred server for protein homology detection and structure prediction (Söding et al., 2005), the *OG1RF_10478* gene could encode a PTS-EIIB component. The three hits with the lowest E-values corresponded to 1) the lactose-specific PTS-EIIB component structure (PDB 3NBM) from *S. pneumoniae* (E-value = 0.015), 2) the cellobiose-specific PTS-EIIB component structure (PDB 4MGE) from *B. anthracis* (E-value = 0.017), and 3) the N,N′-diacetylchitobiose (Chb)-specific PTS-EIIB component structure (PDB 1IIB) from *E. coli* (E-value = 0.054). Nevertheless, unlike most of the PTS-EIIB components, the OG1RF_10478 protein lacks a Cys or His residue at the conserved position usually required for phosphorylation (Leu in OG1RF_10478), suggesting that the OG1RF_10478 protein could be an atypical PTS-EIIB component.

**FIGURE 7:**
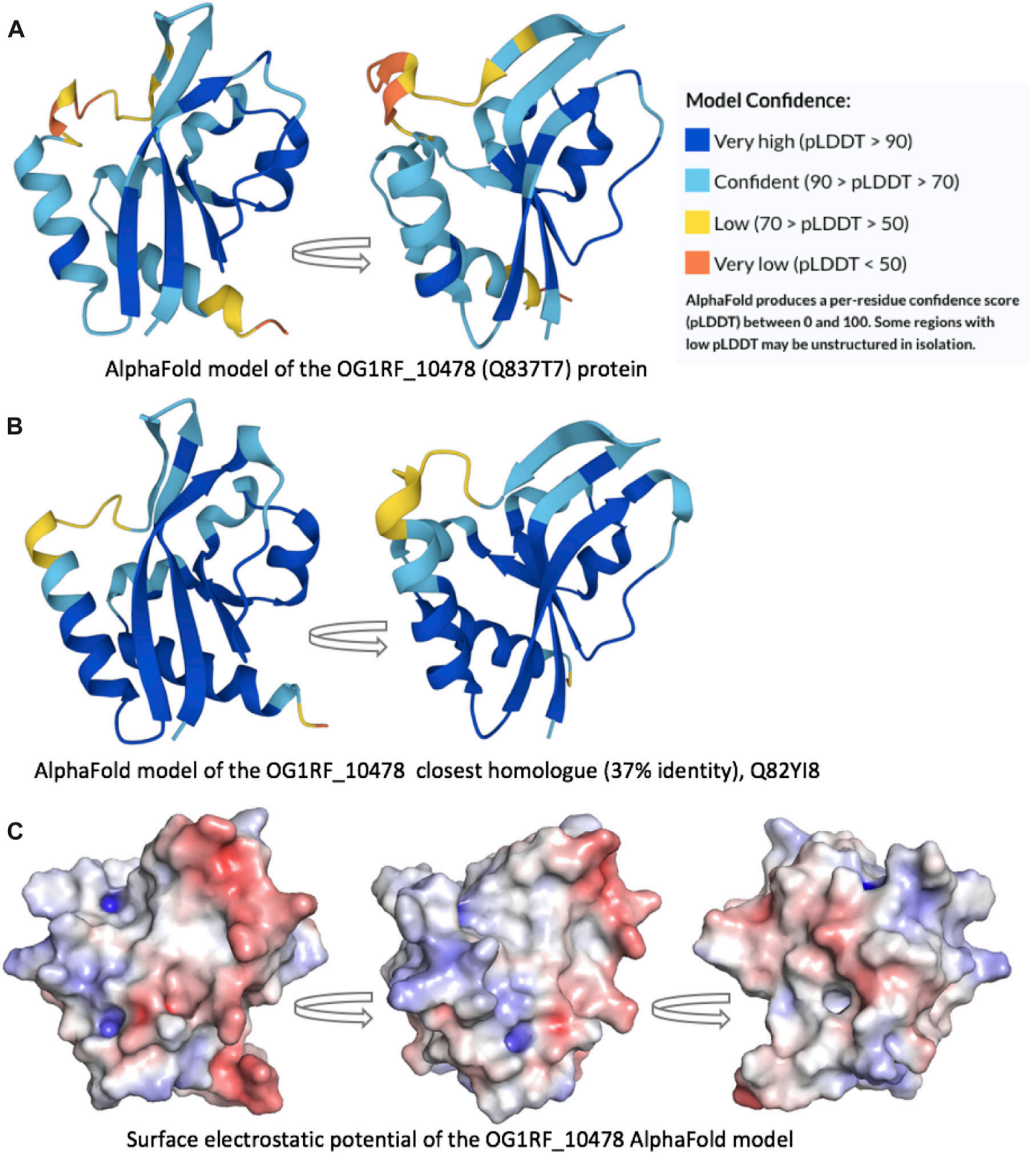
Protein three-dimensional models. AlphaFold models of the **(A)** OG1RF_10478 protein (Q837T7) and **(B)** its closest homologue (Q82YI8; 37% identity). Both models are predicted with high confidence, including the β1-strand end and the short β2-strand. **(C)** Surface electrostatic potential of the OG1RF_10478 model. The arrows represent rotation by 90°.

**FIGURE 8:**
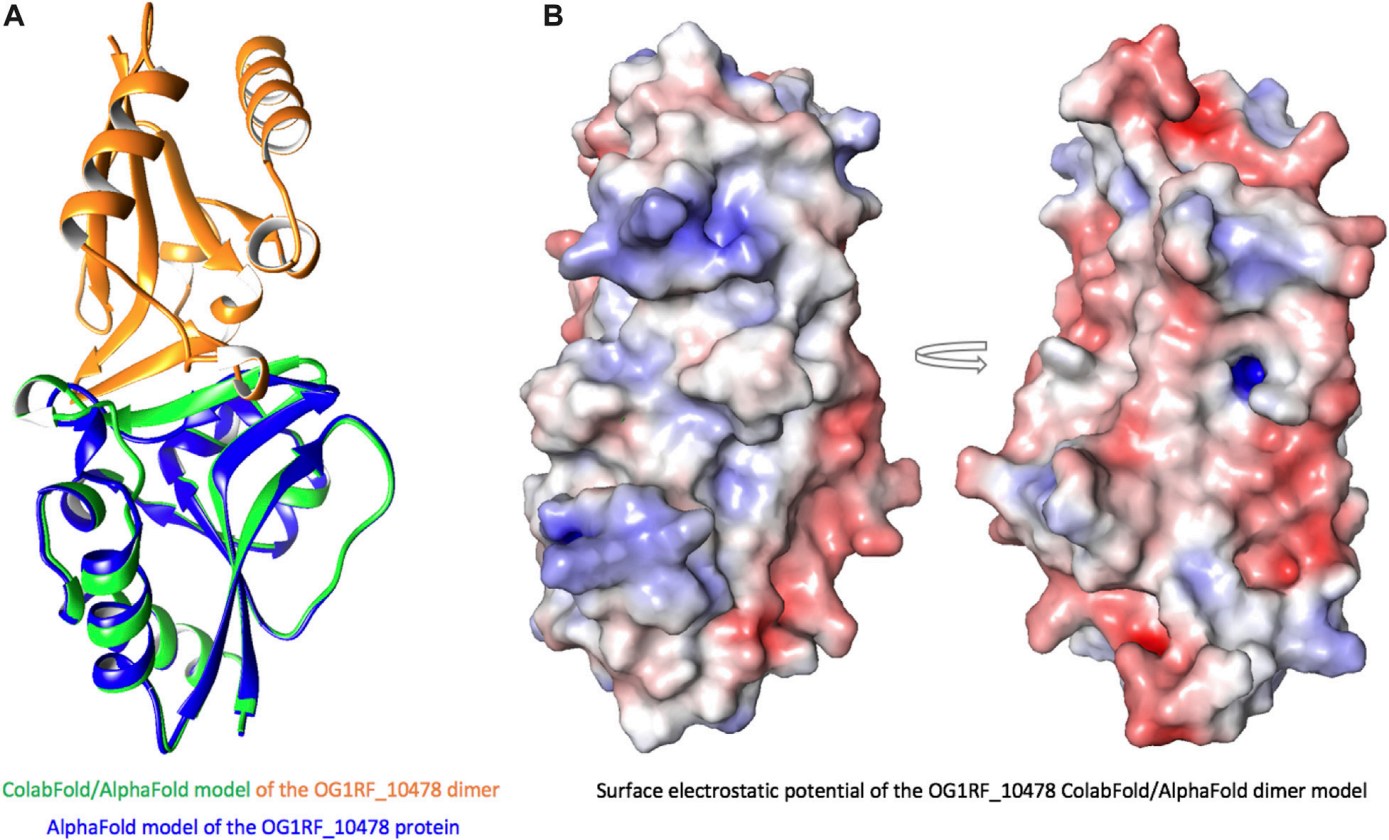
ColabFold/AlphaFold model of the OG1RF_10478 dimer. **(A)** Model of the dimer (green and orange) superposed on the model of the monomer (blue). In the dimer, the β2-strand is longer than in the monomer and forms an interface for intermolecular interactions. **(B)** Surface electrostatic potential of the dimer. The arrow represents rotation by 180°.

To obtain three-dimensional models of the OG1RF_10478 protein (Uniprot Q837T7), we used the AlphaFold Protein Structure Database (Jumper et al., 2021; Varadi et al., 2022) and RoseTTAFold (Baek et al., 2021) due to their ability to accurately predict protein structures. Both computational methods predicted similar OG1RF_10478 structures (2.7 Å RMSD) of the α/β fold class (Figure 7A and Supplementary Figure S3). The main difference between both models was found in the region spanning amino acid residues 10 and 30. Such a region had a low confidence score in the RoseTTAFold model. In the AlphaFold model, the region spanning residues 20 and 30 had also a low confidence score, while the confidently predicted part (residues 10-19) formed an extension of the β1-strand and a short β2-strand, which together with the top part of the β3-strand established a small β-sheet. Noteworthy, the AlphaFold model of an OG1RF_10478 close homologue (37% identity; Uniprot Q82YI8; NCBI Reference WP_002382829.1), which is an uncharacterized protein from *E. faecalis*, exhibits a nearly identical structure (1.1 Å RMSD) and even higher prediction confidence level for the above-mentioned region and in general (Figure 7B). Analysis of the electrostatic surface potential of the OG1RF_10478 model shows that the protein has more negatively than positively charged patches on its surface (Figure 7C; Figure 8), which agrees with its low theoretical isoelectric point (pI: 4.9).

Using the Dali server (Holm, 2022), which performs searches of similar three-dimensional protein structures, we found that the AlphaFold model of OG1RF_10478 is highly similar to 1) structures of PTS-EIIB components (in agreement with the HHpred predictions, see above), and 2) structures of REC domains present in response regulators of TCSs. Specifically, the top ten Dali server hits include three PTS-EIIB components and seven response regulators (Table 2 and Supplementary Figure S4). The REC domain of the response regulators normally includes a conserved Asp residue at the end of the β3-strand that can be phosphorylated by the cognate histidine kinase (Gao et al., 2019). Nevertheless, some REC domains lack such a conserved Asp residue (Maule et al., 2015; Desai and Kenney, 2017). Moreover, although most of the TCS response regulators have an effector domain involved in DNA binding, response regulators made only of the REC domain have been identified (Nguyen et al., 2015). The OG1RF_10478 protein lacks not only the conserved Asp residue (having an Asn residue instead) (Supplementary Figure S5) but also an effector domain, suggesting that OG1RF_10478 might function as a non-canonical response regulator. Nonetheless, based on the sequence identity level between 1) OG1RF_10478 and the top EIIB hit (Chb-specific IIB component from *E. coli*; 19% identity), and 2) OG1RF_10478 and the top response regulator hit (atypical response regulator BaeR from *E. coli*; 15% identity), we hypothesize that protein OG1RF_10478 probably evolved from an EIIB protein rather than from a response regulator.

**TABLE 2:** Top 10 Dali server hits.

Protein	Type	PDB	Z-score	RMSD (Å)	Seq. iden. (%)
N,N′-diacetylchitobiose (Chb)-specific IIB component from *Escherichia coli*	EIIB	2WY2, 1H9C, 1IIB	7.1, 7.0, 6.8	2.7, 2.7, 2.4	19, 19, 19
Antibiotic resistance-associated atypical unphosphorylated dimeric BaeR from *Escherichia coli*	TCS	4B09	6.7	2.7	15
Cellobiose-specific IIB component from *Bacillus anthracis*	EIIB	4MGE	6.5	3.0	13
Biofilm-controlling BfmR from *Acinetobacter baumannii*	TCS	6VBF, 5E3J, 5HM6	6.4, 5.9, 5.9	2.7, 2.8, 2.8	7, 9, 9
Adhesion, biofilm, and virulence-controlling ArlR from *Staphylococcus aureus*	TCS	6IS1	6.4	2.8	12
Fructose-specific EIIB component from *Escherichia coli*	EIIB	4TN5	6.4	2.8	6
Lantibiotic resistance-associated NsTCS from *Streptococcus agalactiae*	TCS	5DCL	6.3	2.8	13
Virulence and antibiotic resistance-controlling VbTCS from *Vibrio parahaemolyticus*	TCS	7E1H	6.2	2.7	10
Colistin resistance-controlling PmrA from *Acinetobacter baumannii*	TCS	7M0S	6.2	2.8	15
Diguanylate cyclase TCS from *Syntrophotalea carbinolica*	TCS	3N53	6.2	2.9	10

When we compared the AlphaFold model of OG1RF_10478 to the structures of the proteins shown in Table 2, we found that the region between residues 10 and 30 in OG1RF_10478 is absent from both the PTS-EIIB components and the response regulators (Supplementary Figure S6). Interestingly, using the ColabFold accelerated prediction of protein structures and complexes software (https://colab.research.google.com/github/sokrypton/ColabFold/blob/main/AlphaFold2.ipynb) (Mirdita et al., 2022), we found that such a region of OG1RF_10478 may function as a homo-dimerization interface (Figure 8). In the predicted dimer model, the dimerization interface consists of the β2-strands from both protomers (the region that is absent in EIIBs and response regulators) and the β2–β2’ interactions promote elongation of the β2-strands in both protomers. In addition to self-dimerization, this region could potentially also serve as a platform for interactions with other proteins.

## 4 Discussion

To generate an adaptive response to fluctuating environmental conditions, the expression of numerous bacterial genes is regulated at the transcriptional level. The promoter sequence of a gene determines significantly the basal transcription initiation frequency. Nevertheless, this frequency may be enhanced and/or reduced by proteins that bind to specific DNA sites, usually close to or even overlapping the binding site of the RNA polymerase (Browning and Busby, 2016; Bervoets and Charlier, 2019). Our previous research established that the *E. faecalis* MafR protein 1) causes genome-wide changes in the transcriptome (Ruiz-Cruz et al., 2016), and 2) functions as a transcription activator (Ruiz-Cruz et al., 2019). Moreover, we have reported that MafR (482 residues) forms dimers in solution and, most likely, binds to its target DNAs as a dimer (Ruiz-Cruz et al., 2018). Now, by qRT-PCR experiments, we have shown that MafR influences positively the transcription of the *OG1RF_10478* gene in bacteria growing exponentially under standard laboratory conditions. Moreover, by primer extension, promoter-reporter fusions, and DNase I footprinting assays, we have identified the *P10478* promoter and demonstrated that MafR stimulates transcription from such a promoter by binding to a DNA site that contains the −35 element (positions −70 to −34; region A). MafR also interacts with a region that is located downstream of the −10 element (positions −2 to +22; region B). These results suggest that MafR might enhance the activity of the *P10478* promoter by recruitment of the RNA polymerase through direct contacts with the sigma factor, as it has been reported for some activators that bind to a site that overlaps the −35 element (Browning and Busby, 2016; Bervoets and Charlier, 2019). Additionally, we speculate that a dimer of MafR might contact the two DNA regions (A and B) and induce conformational changes in the *P10478* promoter, thereby enabling the recognition of the promoter by the RNA polymerase. This mechanism usually involves the binding of the activator to promoters that have non-optimal spacing between the −35 and −10 elements (Browning and Busby, 2016; Bervoets and Charlier, 2019), which is a characteristic of the *P10478* promoter. Regulatory proteins able to repress and activate transcription by differentially modulating local DNA structure within the promoter have been described (Philips et al., 2015). Different from MafR, the pneumococcal Mga*Spn* and PclR activators (also members of the Mga/AtxA family) bind to a DNA site that is located upstream of the main promoter elements. Mga*Spn* recognizes a site located between positions −99 and −60 of the *P1623B* promoter (Solano-Collado et al., 2013) and PclR recognizes a site located between positions −169 and −68 of the *PpclA* promoter (Moreno-Blanco et al., 2022). Activators that bind to a DNA site located upstream of the promoter typically recruit the RNA polymerase by interacting with the α-subunit (Browning and Busby, 2016; Bervoets and Charlier, 2019).

MafR-mediated transcriptional activation has also been reported for the *P11486* and *P12294* promoters (Ruiz-Cruz et al., 2019). In both cases, MafR binds to a DNA site that contains the −35 promoter element, as well as regions of potential bendability. Furthermore, studies on the *Pma* promoter of the *mafR* gene have shown that MafR interacts with a site that is located 1) upstream of the main promoter elements (positions −104 to −69), and 2) adjacent to the peak of a potential intrinsic curvature (Ruiz-Cruz et al., 2018). However, the function (if any) of this interaction remains unknown. As shown in Figure 6, the DNA sites recognized by MafR on the *P10478*, *P11486*, *P12294*, and *Pma* promoters have a low sequence identity, which suggests that MafR could recognize structural features in its target DNAs rather than a specific nucleotide sequence. Several studies support that recognition of local DNA conformations (shape readout mechanism) contributes to the DNA-binding specificity of the regulators that constitute the Mga/AtxA family (Hadjifrangiskou and Koehler, 2008; Hause and McIver, 2012; Solano-Collado et al., 2013; Ruiz-Cruz et al., 2018). In the case of the pneumococcal Mga*Spn* regulator, *in vitro* DNA binding studies have shown that it binds to linear double-stranded DNAs with little or no sequence specificity (Solano-Collado et al., 2013). Moreover, Mga*Spn* has a preference for AT-rich DNA sites (Solano-Collado et al., 2016) and for DNA regions that contain a potential intrinsic curvature (Solano-Collado et al., 2013).

What could be the role of the OG1RF_10478 protein? We postulate that it could have a regulatory function. This suggestion is based on the function of proteins whose three-dimensional structure is similar to the OG1RF_10478 model predicted with AlphaFold (this work). First, we have found significant structural similarity between OG1RF_10478 and some PTS-EIIB components. However, different from such components, OG1RF_10478 lacks a conserved Cys or His residue at the position usually required for PTS-mediated phosphorylation. This finding suggests that the putative regulatory role of OG1RF_10478 could be achieved by protein-protein interactions, as it has been shown for some unphosphorylated PTS-EIIB components that, either as distinct proteins or fused to another PTS component, control the activity of particular transcription regulators by interacting with them (Deutscher et al., 2014; Galinier and Deutscher, 2017). In line with our hypothesis, the ColabFold software (Mirdita et al., 2022) predicts that a region of OG1RF_10478 could serve as a platform for interactions with other proteins (this work). Moreover, it has been pointed out that PTS-mediated regulation by protein-protein interactions is likely more frequent than by phosphorylation (Deutscher et al., 2014). Second, we have found structural similarity between OG1RF_10478 and the REC domain of some TCS response regulators. Nevertheless, unlike the majority of such regulators (Gao et al., 2019), OG1RF_10478 lacks an effector domain. Moreover, the REC domain typically has a conserved Asp residue that is missing in the OG1RF_10478 protein. Phosphorylation of this residue by the cognate histidine kinase is critical for the activity of the response regulator (Gao et al., 2019). Therefore, OG1RF_10478 could be considered a non-canonical response regulator that consists of a stand-alone REC domain without the conserved Asp residue. Numerous studies on TCSs have identified different types of non-canonical response regulators. For instance, there are regulators that only have a REC domain with the conserved Asp residue, the so-called single-domain response regulators (Jenal and Galperin, 2009). The phosphorylated forms of some of these regulators play key regulatory roles when they interact with other proteins. In these cases, the phosphoryl group can stay on the REC domain or be transferred to another protein (Galperin, 2006; Jenal and Galperin, 2009; Nguyen et al., 2015). Furthermore, there are response regulators that lack the conserved Asp residue of the REC domain and then regulation of their activity is not via phosphorylation. To regulate the activity of this class of regulators, different strategies have been identified (Maule et al., 2015; Gao et al., 2019). Examples of response regulators whose unphosphorylated and phosphorylated forms have distinct regulatory roles have also been reported (Desai and Kenney, 2017).

In conclusion, our present work demonstrates that MafR activates the transcription of the *OG1RF_10478* gene by binding to a DNA site that contains the −35 element of the *P10478* promoter. This DNA site has a low sequence identity with the MafR binding sites identified previously. Moreover, our study shows that the predicted three-dimensional model of the OG1RF_10478 protein is similar to the structure of some PTS-EIIB components and to the structure of the REC domain of some TCS response regulators. Based on these similarities, we propose that OG1RF_10478 could be a regulatory protein.

## Data Availability

The original contributions presented in the study are included in the article/Supplementary Material, further inquiries can be directed to the corresponding authors.

## Abbreviations

BHI, Brain Heart Infusion; OD_650_, optical density at 650 nm; PRD, PTS regulation domain; PTS, phosphotransferase system; REC domain, receiver domain; TCS, two-component signal transduction system; TY, tryptone-yeast extract.
