# Supplementary material for: Promoter DNA recognition by the Enterococcus faecalis global regulator MafR
Source: Front Mol Biosci. 2023 Dec 13;10:1294974. doi: 10.3389/fmolb.2023.1294974 (PMC10773906; doi:10.3389/fmolb.2023.1294974)
Supplement: Supplementary file 1 [file DataSheet1.PDF]

SUPPLEMENTARY INFORMATION

Promoter DNA recognition by the *Enterococcus faecalis* global regulator MafR

Ana Moreno-Blanco[1,3], Radoslaw Pluta[2], Manuel Espinosa[1], Sofía Ruiz-Cruz[1,4,*] and Alicia Bravo[1,*]

[1] Centro de Investigaciones Biológicas Margarita Salas, Consejo Superior de Investigaciones Científicas (CSIC), Madrid, Spain

[2] Institute for Research in Biomedicine (IRB Barcelona), The Barcelona Institute of Science and Technology, Barcelona, Spain

[3] **Present address:** Servicio de Microbiología del Hospital Universitario Ramón y Cajal, Instituto Ramón y Cajal de Investigación Sanitaria (IRYCIS) and CIBERINFEC, Madrid, Spain

[4] **Present address:** Facultad de Ciencia y Tecnología, Departamento de Bioquímica y Biología Molecular, Universidad del País Vasco, Leioa, Vizcaya, Spain

***Corresponding authors**
E-mail addresses: sofia.ruizcruz@ehu.eus (Sofía Ruiz-Cruz); abravo@cib.csic.es (Alicia Bravo)
Centro de Investigaciones Biológicas Margarita Salas, Consejo Superior de Investigaciones Científicas (CSIC). Ramiro de Maeztu 9, 28040 Madrid, Spain
Tel: +34 918373112 (Alicia Bravo)

Table S1: Relative expression of the *OG1RF_10478* gene in OG1RF (strain A) and OG1RFΔ*mafR* (strain B)

Strain	cDNA	C_T *10478* [(1)]	C_T *recA* [(2)]	$2^{-\Delta CT}$ [(3)]	Mean ± sd [(4)]	FC[(5)]	P-value
	1	18.65	19.45	1.742			
A	2	18.66	18.57	0.944	1.337 ± 0.399		
	3	19.73	20.14	1.325			
						9.62	0.049
	1	23.11	19.66	0.091			
B	2	21.09	19.09	0.249	0.139 ± 0.096		
	3	23.30	19.59	0.077			

Table S2: Relative expression of the *OG1RF_10478* gene in OG1RFΔ*mafR* harbouring plasmid pDLF*mafR* (strain C) and OG1RFΔ*mafR* harbouring plasmid pDLF (strain D)

Strain	cDNA	C_T *10478* [(1)]	C_T *recA* [(2)]	$2^{-\Delta CT}$ [(3)]	Mean ± sd [(4)]	FC[(5)]	P-value
	1	22.30	18.96	0.099			
C	2	22.33	19.11	0.107	0.107 ± 0.008		
	3	22.22	19.12	0.116			
						2.73	0.002
	1	23.01	18.26	0.037			
D	2	23.15	18.36	0.036	0.039 ± 0.004		
	3	22.71	18.21	0.044			

For each strain, total RNA was isolated from three independent bacterial cultures. cDNA (random primers) was synthesized from each RNA preparation. From each cDNA sample, three PCRs per gene were performed.

(1) Mean C_T from the three PCRs for the *OG1RF_10478* gene

(2) Mean C_T from the three PCRs for the *recA* gene (internal control gene)

(3) $\Delta C_T = C_T$ *OG1RF_10478* gene - C_T *recA* gene

(4) Mean ± standard deviation of the $2^{-\Delta CT}$ values

(5) Fold change in expression of the *OG1RF_10478* gene

P-values were calculated using the Student´s *t*-test (paired, two-tailed)

See the graphical representation in Figure 2

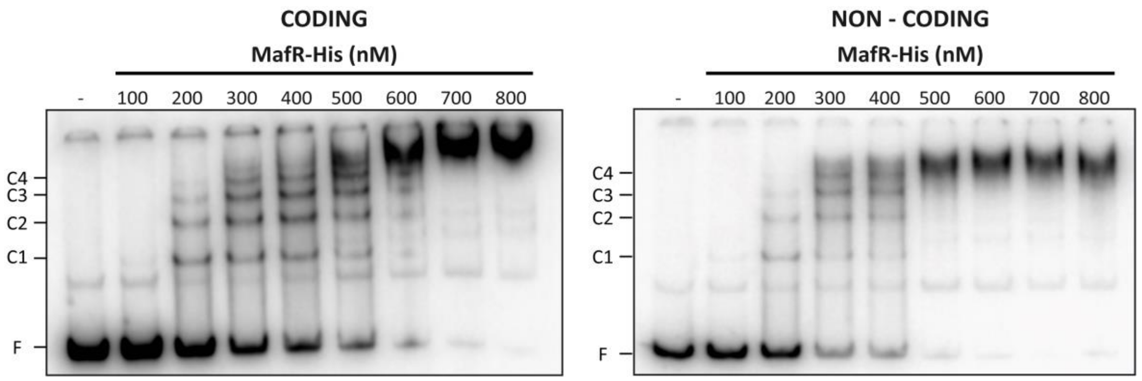

Figure S1. EMSA experiments. Binding of MafR-His to the 266-bp DNA fragment (coordinates 498475 to 498210 of the OG1RF genome), which contains the *P10478* promoter. The DNA fragment (4 nM) (radioactively labelled either at the 5´-end of the coding strand or at the 5´-end of the non-coding strand) was incubated with the indicated concentrations of protein. Free and bound DNAs were separated by native polyacrylamide (6%) gel electrophoresis. Labelled DNA was visualized using a Fujifilm Image Analyser (FLA-3000). Bands corresponding to free DNA (F) and several protein-DNA complexes (C1 to C4) are indicated.

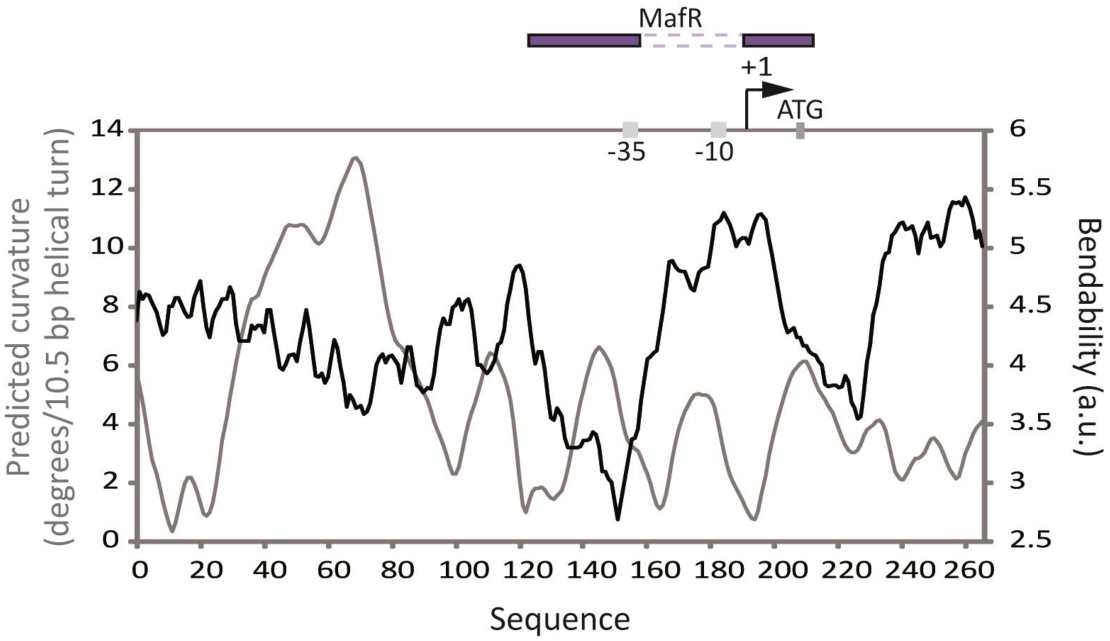

Figure S2. Bendability/curvature propensity plot of the 266-bp DNA fragment (coordinates 498475 to 498210 of the OG1RF genome) according to the bend.it server (pongor.itk.ppke.hu/dna/bend_it.html) (Vlahovicek et al., 2003). The location of the -35 and -10 elements of the *P10478* promoter is indicated. The transcription start site (+1 position) and the translation start codon (ATG) of the *OG1RF_10478* gene are indicated. The purple rectangles represent the regions recognized by MafR (defined by DNase I footprinting assays).

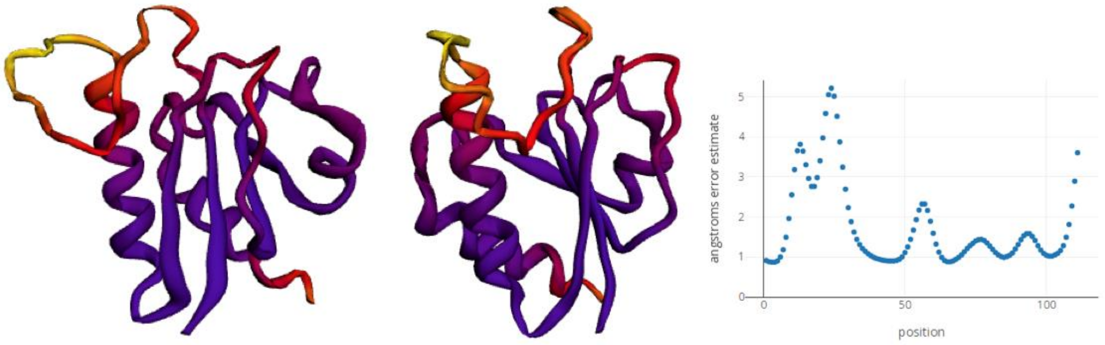

Figure S3. RoseTTAFold-predicted OG1RF_10478 three-dimensional model. Two snapshots rotated by 90 degrees and a plot of confidence/error score in angstroms for each residue. The colour scale goes from violet (low error) to yellow (high error).

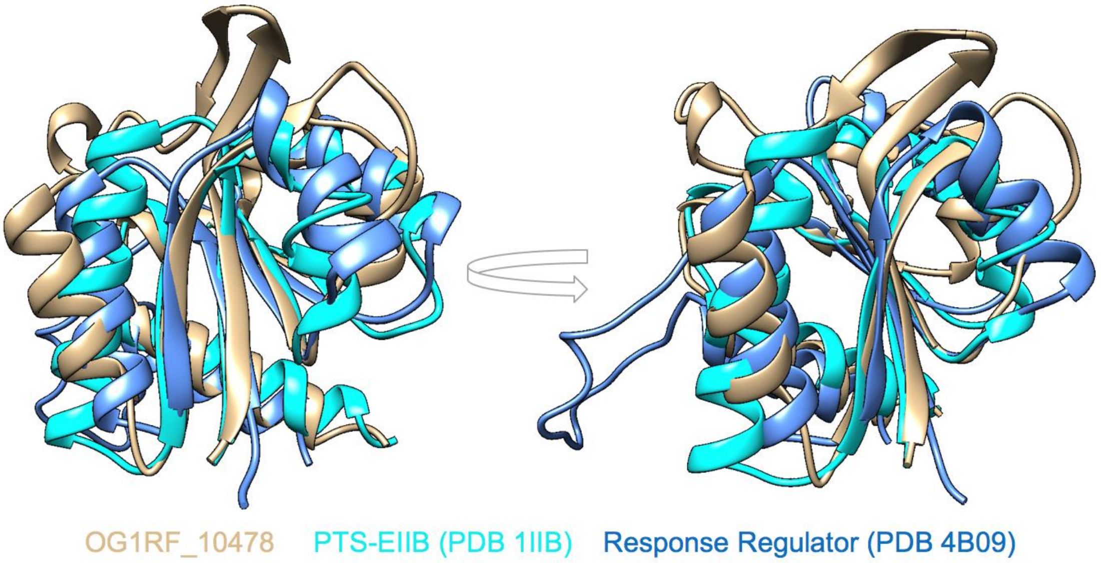

Figure S4. Superposition of the OG1RF_10478 AlphaFold model and crystal structures of the top PTS-EIIB (PDB 1IIB; cyan) and response regulator (PDB 4B09; blue) Dali hits. The arrow represents rotation by 90 degrees.
PDB 1IIB: N,N'-diacetylchitobiose (Chb)-specific IIB component from *Escherichia coli*
PDB 4B09: Antibiotic resistance-associated atypical unphosphorylated dimeric BaeR from *E. coli*.

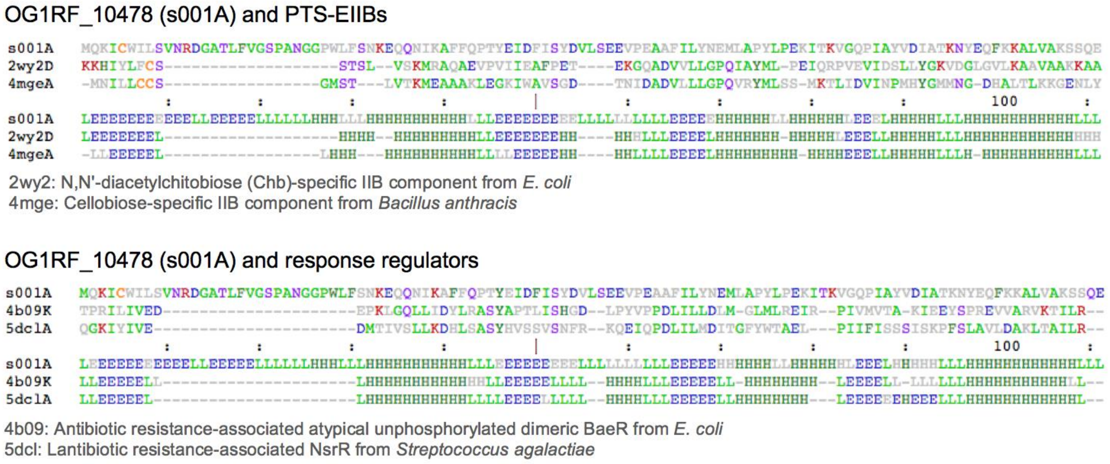

Figure S5. Structure-based sequence alignment of OG1RF_10478 (s001A) and PTS-EIIBs (PDB: 2WY2 and 4MGE) and response regulators (PDB: 4B09 and 5DCL). Sequences are shown in the pairwise Dali-alignment to OG1RF_10478 (shown as s001A). Uppercase highlight structurally equivalent positions with OG1RF_10478. The most frequent amino acid type is coloured in each column. The upper lines in both alignments present the amino acid sequences and the lower lines show the secondary structure (H/h: helix, E/e: strand, L/l: coil). For clarity, inserted segments relative to the OG1RF_10478 are hidden.

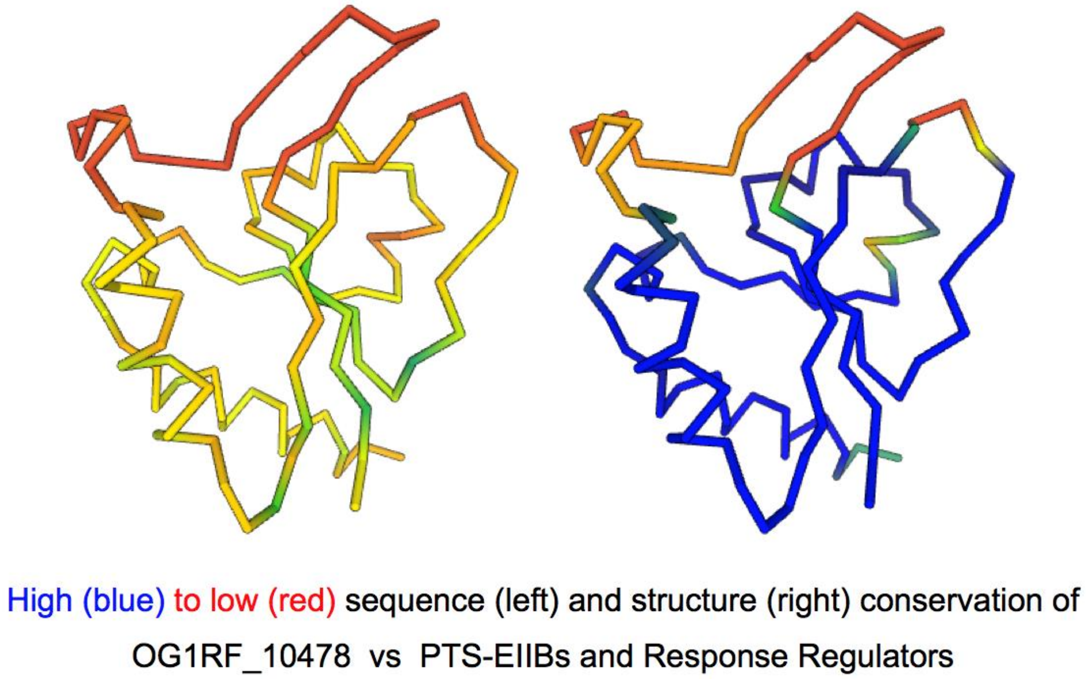

Figure S6. Sequence and structure conservation of the OG1RF_10478 AlphaFold three-dimensional model compared to PTS-EIIBs and response regulators. The AlphaFold model versus most similar experimental structures found by DALI server. Proteins are shown in ribbon representation. From blue to red (high to low) sequence (*left*) and structure (*right*) conservation.
